# New Trends in Separation Techniques of Lithium Isotopes: A Review of Chemical Separation Methods

**DOI:** 10.3390/ma16103817

**Published:** 2023-05-18

**Authors:** Silviu-Laurentiu Badea, Violeta-Carolina Niculescu, Andreea-Maria Iordache

**Affiliations:** National Research and Development Institute for Cryogenic and Isotopic Technologies, 4th Uzinei Street, 240050 Râmnicu Vâlcea, Romania; silviu.badea@icsi.ro (S.-L.B.); andreea.iordache@icsi.ro (A.-M.I.)

**Keywords:** lithium isotopes, crown ethers, electromigration, ion exchange, MC-ICP-MS

## Abstract

In terms of isotopic technologies, it is essential to be able to produce materials with an enriched isotopic abundance (i.e., a compound isotopic labelled with ^2^H, ^13^C, ^6^Li, ^18^O or ^37^Cl), which is one that differs from natural abundance. The isotopic-labelled compounds can be used to study different natural processes (like compounds labelled with ^2^H, ^13^C, or ^18^O), or they can be used to produce other isotopes as in the case of ^6^Li, which can be used to produce ^3^H, or to produce LiH that acts like a protection shield against fast neutrons. At the same time, ^7^Li isotope can be used as a pH controller in nuclear reactors. The COLEX process, which is currently the only technology available to produce ^6^Li at industrial scale, has environmental drawbacks due to generation of Hg waste and vapours. Therefore, there is a need for new eco-friendly technologies for separation of ^6^Li. The separation factor of ^6^Li/^7^Li with chemical extraction methods in two liquid phases using crown ethers is comparable to that of COLEX method, but has the disadvantages of low distribution coefficient of Li and the loss of crown ethers during the extraction. Electrochemical separation of lithium isotopes through the difference in migration rates between ^6^Li and ^7^Li is one of the green and promising alternatives for the separation of lithium isotopes, but this methodology requires complicated experimental setup and optimisation. Displacement chromatography methods like ion exchange in different experimental configurations have been also applied to enrich ^6^Li with promising results. Besides separation methods, there is also a need for development of new analysis methods (ICP-MS, MC-ICP-MS, TIMS) for reliable determination of Li isotope ratios upon enrichment. Considering all the above-mentioned facts, this paper will try to emphasize the current trends in separation techniques of lithium isotopes by exposing all the chemical separation and spectrometric analysis methods, and highlighting their advantages and disadvantages.

## 1. Introduction

Lithium-6 (^6^Li) and lithium-7 (^7^Li), with the latter being far more abundant (95.15%), are the two stable isotopes of lithium occurring in Earth’s lithosphere. A fractionation of lithium isotopes appears during a variety of geological processes [1,2]. Such processes include mineral formation (by chemical processes like precipitation and ion exchange). Lithium 6 isotope (^6^Li) is sometimes preferred over ^7^Li, as the Li^+^ ions exchange magnesium or iron in octahedral locations of the clays, which leads to an enrichment of ^6^Li in geological processes [3,4].

In laboratory and technological processes, ^6^Li is an important isotope for tritium production via bombardment with neutrons, therefore several techniques for its separation have been developed in the past. Historically, COLEX processes have been used in the past (in the 1950s and 1960s) for lithium isotope separations based on greater affinity of ^6^Li over ^7^Li for the element mercury. The working principle of the COLEX technology is based on the fractionation of ^6^Li between a lithium amalgam and a lithium hydroxide solution, as the amalgam and lithium hydroxide solution come into contact. At industrial scale, the COLEX technology uses this principle in sequential stages, by passing an aqueous lithium hydroxide flowing up and a counter-flow of lithium-mercury amalgam flowing down. At the top of the column, the lithium hydroxide solution is electrolyzed to liberate the depleted ^7^Li fraction, while at bottom of the column, the ^6^Li-enriched fraction is separated from the amalgam, and the metallic Hg is recovered and reused in the process. The main advantage of COLEX technology is its minimal production costs, which favours the industrial scale production of enriched lithium. Nevertheless, nowadays its technical (decomposition of the amalgam, high energy consumption) and environmental (generation of toxic Hg vapours, large quantities of Hg-containing waste) drawbacks make this technology obsolete [5]. Additionally, other methods like vacuum distillation were tested in the 1950s [6], but this technology was not applied extensively, possibly due to high energy consumption. More recently, a variety of methods have been developed for lithium isotope separations [7,8,9,10]. Based on their working principles, these methods can be classified into three main groups: (1) chemical exchange methods in two liquid phases, (2) electrochemical exchange methods, and (3) displacement chromatography methods (see Table 1). Chemical exchange methods in two liquid phases employ crown ethers as extractants that can be also used in combination with ion liquids as extraction solvents [11,12]. The crown ethers have the capacity to separate lithium from other alkaline ions [13] as well to differentiate between ^6^Li and ^7^Li isotopes. The ion liquids can separate lithium from other alkaline ions and complex metal matrices like lithium battery waste [14], but they cannot solely differentiate between ^6^Li and ^7^Li, and they must be used in combination with crown ethers [11]. The main advantage of chemical exchange methods in two liquid phases is the separation factor of lithium isotopes (comparable with COLEX technology), but this comes with the drawbacks of very high costs for these reagents, as well their relative toxicity. Electrochemical exchange methods like electromigration and electrodialysis are more eco-friendly [15], but they result in relatively low separation factors of lithium isotope and they require complex experimental methodologies. Displacement chromatography methods have the advantages of involving reusable ion exchange columns (and thus are eco-friendly), but the obtained separation factors are relatively low.

The objective of this paper is to highlight the current trends in separation techniques for lithium isotopes, emphasizing the chemical separation methods and spectrometric analysis methods.

## 2. Challenges in Development of Separation Methods for Lithium Isotopes Using Chemical Exchange Methods in Two Liquid Phases

The paradigm of this type of separation method is that the lithium compounds will move between the two immiscible stages if the solvation changes slightly. Research indicates that there are not many chemical exchange methods for isotope separation. Gas-liquid systems make up most of the systems that have been reported so far. Because there are no suitable gaseous lithium compounds for such isotope exchange [16], the liquid–liquid partition must be used for lithium isotope separation. In the single-stage liquid–liquid partition of Li isotopes, the separation coefficient is defined according to the Equation (1).

(1) ∝=L6iL7i1L6iL7i2

where (^6^Li/^7^Li) represents the isotope ratio, and the subscripts 1 and 2 denote the two phases or two compartments used in the separation tests. An alternative form of separation coefficient was used in the Li isotope fractionation tests, which is usually called ^7^Li fractionation (expressed as δ ^7^Li. The commonly expressed stable isotope ratio of a given compound is the deviation δ [‰] from an international standard [17], similar to carbon and other isotopes according to the Equation (2).

(2) δLi7=L7iL6isampleL7iL6iL-SVEC - 11000

where L-SVEC represents an international standard with a well-defined isotopic composition (^7^Li/^6^Li ratio = 12.33 ± 0.03).

### 2.1. Separation Using Crown Ethers

Several studies have confirmed the good separation effect of macrocyclic compounds like crown ether (CE) and its derivatives on Li isotopes. The principle of this separation is that the solvation environments of the cations are quite different when a metal salt is distributed between an immiscible solvent containing a macrocyclic compound and an aqueous solution.

Due to their unique cavity structures and size effect, the separation factor of ^6^Li/^7^Li (the α value) by crown ethers is comparable to that of the Li amalgam method. However, the relatively weak interaction of crown ethers with Li^+^ leads to a low distribution coefficient of Li (DLi, 10^−2^–10^−5^), especially for crown ethers with small cavities such as 12-crown-4 (12C4), B12C4, whereas these crown ethers generally demonstrate much higher α values. Another challenge is the loss of CE molecules during the extraction procedure. Several studies have reported the separation of ^6^Li/^7^Li isotopes using solvent extraction with crown ethers [18,19]. The selection of crown ethers must consider their cavity sizes that must closely match that Li^+^ ionic diameter [20,21]. Furthermore, it was shown that due to the dipole–ion interactions between the donor O atoms and Li^+^, the cavity sizes of free 12C4, 15C5, and 18-Crown-6 (18C6) crown ethers decrease upon their coordination with Li^+^ ion [22]. Therefore, crown ethers with large cavities such 15C5, 18C6, and DC18C6 could be chosen for their higher distribution coefficient.

For example, Nishizawa et al. investigated the Li isotopic effects of 12C4, B15C5, and dicyclohexano-18-crown-6 (DC18C6) with solvent extraction and found that the smaller-cavity-sized crown ethers have a greater separation factor [19]. The separation factor for 12C4-CHCl_3_/LiI-H_2_O system was reported to be 1.057 at 0 °C, while D_Li_ was only 2.0 × 10^−5^, lower than that of B15C5 and DC18C6 (10^−2^–10^−3^). Due to the low distribution of Li for 12C4 crown ether, the 15C5 crown ether and its derivates have been used in recent studies. Some studies tried the development of covalently grafted functional materials, which can significantly reduce the loss of CE molecules. For example, Liu et al. (2016) used grafted NH_2_-B15C5 on mesoporous silica SBA-15 and investigated the adsorptive separation behaviour of ^6^Li/^7^Li [23].

**Figure 1:**
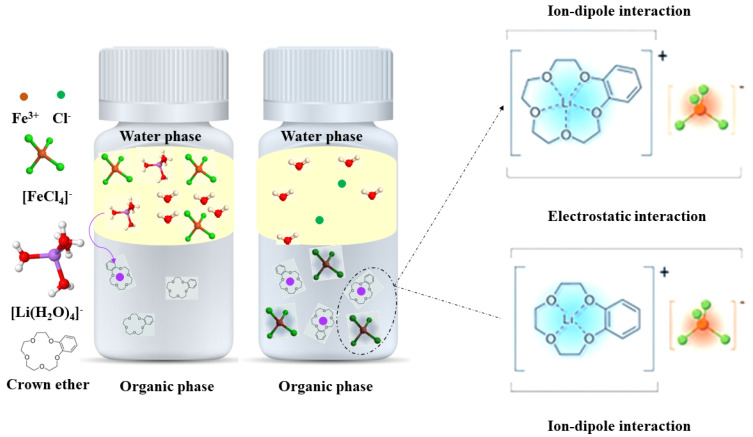
Overview of the ion–pair strategy for ^6^Li separation using crown ethers B15C5 or B12C4 and [FeCl_4_]^−^ as counter anion. Adapted from [24].

A more recent strategy (called ion–pair strategy) was developed by Cui et al. to create an efficient phase transfer of Li^+^ by crown ethers. In this system, FeCl_3_ salt, known as a strong Lewis acid, was introduced for Cl^−^ binding to form [FeCl_4_]^−^ anion, and B12C4 or B15C5 acted as Li^+^ receptor [24]. The presence of tetrachloridoferrate ([FeCl_4_]^−^) as counter anion was presented, which helps to overcome the Hofmeister bias and facilitates the efficient transfer of Li+ from extremely hydrophilic chlorides (see Figure 1).

Upon the coupling effects of electrostatic and ion–dipole interactions, this new strategy showed an unprecedented distribution of Li^+^ in the solvent extraction process. An exceptionally high D_Li_ of 54 was achieved by Cui et al., 2021 for benzo-15-crown-5 (B15C5), surpassing those of solvent extraction from solely Li salt aqueous solution. In this study, the maximum ^6^Li/^7^Li separation factors (α) of 1.038 and 1.049 were obtained for B15C5 and benzo-12-crown-4 (B12C4) in dichloroethane at 273 K [24].

### 2.2. Challenges in Development of Separation Methods for Lithium Isotopes Using Electrochemical Exchange Methods

#### 2.2.1. Challenges in Development of Separation Methods for Lithium Isotopes with Electromigration

Electrochemical separation of lithium isotopes through the difference in migration rates between ^6^Li and ^7^Li is one of the green and promising alternatives for the separation of lithium isotopes [15]. According to media of lithium transferring, it can be divided into molten salt methods, aqueous solution methods, and organic solvent methods. However, due to the problems of corrosion resulting from high-temperature molten salt, the last two above-mentioned methods are preferred. Electromigration in aqueous solution system has good reproducibility; the electrode reactions take place in aqueous solution, so it is easy to realize multistage separation. However, due to the strong hydration of lithium ions which affects their migration, it is difficult to achieve a high separation effect. Therefore, the organic solvent method using ionic liquid, diethyl carbonate, and crown ethers was employed in recent studies. Wang et al. employed electromigration from lithium-loaded organic phase aqueous solution using ionic liquids (1-octyl-3-methylimidazolium bis(trifluoromethanesulfonyl)imide ([C_8_MIm][NTf_2_]), crown ethers (benzo 15-crown-5) and the organic sulfonimide lithium salt (Li[NTf_2_]) [25]. The maximum isotope fractionation of ^7^Li upon electromigration was found to be −21.5‰ vs. L-SVEC, fractionation that is attributable to the differences in the dissociation process of ^6^Li vs. ^7^Li ions-crown ethers complex [25]. In their next study, by also employing a new crown ether (4-nitrobenzo-15-crown-5) and a new ionic liquid (1-butyl-3-methylimidazolium bis[(trifluromethyl) sulfonyl]imide ([BMIm][NTf_2_]), Wang et al. compared two experimental approaches: I. system lithium ions–ionic liquid–crown ether organic solution–aqueous solution, and II. aqueous solution–organic solution–aqueous solution system, for the separation of lithium isotopes using electromigration [15]. It was found that there are multiple roles for crown ether: phase transfer, selective chelation, dehydration, and retention during electromigration. Wang et al. found a fractionation of Li isotopes, as the δ^7^Li values increase from 15‰ to 16.1, as the concentrations of crown ether increase from 0 to 0.2 mol/L [15].

Very recently, in their last electromigration study, Wang et al. tested different catholytes (HCl, NH_3_·H_2_O, NH_4_Cl and NH_4_HCO_3_) during separation of lithium isotopes with electromigration by using a lithium salt aqueous solution–organic solution–aqueous solution system [26]. In this study, the highest separation coefficient of 1.674 was obtained using a solution of 0.1 mol/L NH_4_Cl as catholyte [26]. Overall, the above-mentioned studies shown the potential of electromigration methodologies in eco-friendly separation of lithium isotopes.

#### 2.2.2. Challenges in Development of Separation Methods for Lithium Isotopes Using Electrodialysis Methods

**Figure 2:**
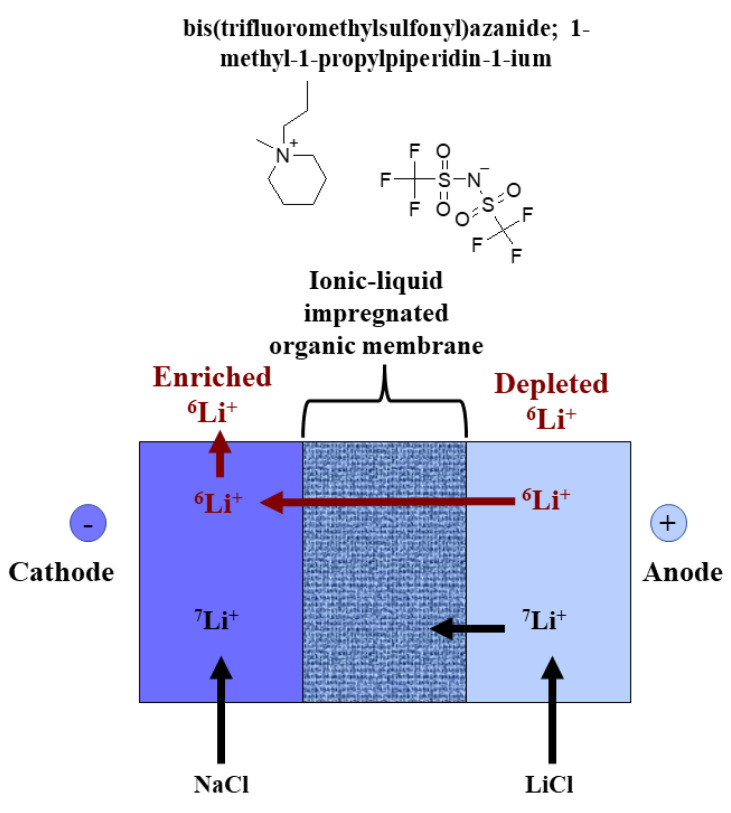
Separation of lithium isotopes with electrodialysis using an impregnated organic membrane with PP13-TFSI ionic liquid. The formula of ionic liquid was generated using InChI (Computed by InChI 1.0.6-PubChem release 7 May 2021) within the software Freeware ACD/ChemSketch 2021 2.0 version (Advanced Chemistry Development, Inc., Toronto, ON, Canada). Adapted from [27].

Lithium ions can move by electrodialysis through certain Ionic-Liquid-i-OMs between the cathode and the anode in lithium solutions. The principle is that since the ionic mobility of ^6^Li ions is greater than that of ^7^Li ions, ^6^Li can be enriched on the cathode side of a cell (see Figure 2).

Hoshino and Terai used PP13-TFSI (N-methyl-N-propylpiperidium bis(trifluoromethanesulfonyl)imido) ionic liquid to impregnate a highly porous Teflon film, while both surfaces of the Ionic-Liquid-i-Organic Membrane were covered by a nafion 324 overcoat or a cation exchange membrane [27]. Hoshino and Terai applied this membrane in an electrodialysis experiment [27]. It was found that the ^6^Li isotope separation factor with electrodialysis using highly porous Teflon film of 3 mm thickness was larger than that using highly porous Teflon film of 1–2 mm thickness [27]. In particular, ^6^Li isotope separation factor using the Teflon of 3 mm thickness was about 1.15 at 0.01% of Li movement rat; on the other hand, using the Teflon of 1–2 mm thickness it was about 1.05 [27].

Besides the use of ionic liquids, the separation of Li isotopes with electrodialysis also can be achieved using lithium solid ceramics. Shin-mura et al. developed a method for separation of lithium isotopes using La_0.57_Li_0.29_TiO_3_ as a solid ceramic electrolyte in the temperature range 298.15–323.15 K [28]. With the respect to the rate of transfer of ^6^Li and ^7^Li into water with electrodialysis, an Arrhenius-type dependence of temperature was concluded [28]. Overall, this study showed that the apparent activation energy of ^7^Li is approximately 5% higher than that of ^6^Li, and this is attributable to the quantum effect [28]. Nevertheless, ^6^Li isotope separation factor was not calculated in the above-mentioned paper and further studies are needed. Furthermore, the lithium solid ceramics can be used to separate lithium ions from sodium and potassium, and Ounissi et al. used lithium composite membrane to separate lithium from sodium ions [29] with electrodialysis, but the selectivity coefficient of Li^+^ over Na^+^ was recorded to 112.3, a value which is higher than the selectivity coefficients previously reported in other electrodialysis methodologies [30]. Nevertheless, cation-exchange resins methods are usually preferred as preconcentration steps employed for the separation of lithium from other alkaline ions.

### 2.3. Challenges in Development of Separation Methods for Lithium Isotopes Using Displacement Chromatography Methods

#### 2.3.1. Inorganic Microstructure Methods

There are just a few studies involving separation of lithium isotopes using displacement chromatography based on inorganic microstructures. Separation of lithium was investigated using an ion exchange column filled with MnO_2_ and the exchange capacity of MnO_2_ oxide was calculated to 0.5 meq/g [7]. In this study, the MnO_2_ phase was enriched in ^6^Li and the enriched fraction was eluted using CH_3_COONa with concentration of 2 M. The separation factor (α) of ^6^Li was 1.026 [7], a value which is lower compared with the values obtained using equilibrium phase exchange with crown ethers. More recently, Ishikawa et al., 2017 [31] measured isotopic ratios ^7^Li/^6^Li for effluent fractions from a biphasic zeolite column ([Li_0.08_(NH_4_)_0.92_]A and [Li_0.33_(NH_4_)_0.67_]A hydrates). The study shown the accumulation of 6Li in the zeolite proceeded by a mechanism of differential elution of ^7^Li from the biphasic zeolite.

#### 2.3.2. Ion-Exchange Methods Using Resin

Another type of displacement chromatography method is the separation of lithium isotopes using ion-exchange resin. Many studies on lithium isotope separation have been conducted using different types of cation-exchange resins. Nevertheless, due to natural abundance of sodium ions, a preconcentration step is needed to separate lithium from others alkaline ions prior to separation of lithium isotopes [32]. For example, Karami et al. used an AG 50W-X8 cation-exchange resin (200–400 mesh, protonated form) packed in a 20 cm × 2 cm I.D. glass column to separate lithium from sodium and potassium ions [33]. Using 0.1 M HCl solution as eluent, the separation and recovery efficiency for lithium ions was recorded to 99.24%, which indicates a very good separation [33].

Taylor and Urey were the first to use and report on the cation-exchange method for Li isotope separation in 1937 and since then several other studies were performed using cation-exchange methods for lithium isotope separation [34]. For example, lithium isotope isolation of lithium lactate in dimethyl sulfoxide (DMSO) and water–acetone mixed-solvent media with ion-exchange displacement chromatography was also investigated by Oi et al. [35] and a convex function of the solvent mixing ratio was observed in the single-stage separation factor. The single-stage separation factor had its highest value of 1.00254 at water:DMSO = 25:75 *v*/*v* and 1.00182 at water:acetone = 75:25 *v*/*v* [35]. In order to improve the separation of lithium isotopes, a sulfonated-type cationic exchange resin with higher cross-linkage of 50% (obtained by adding styrene and divinylbenzene in a proportion 1:1) higher than those commercially available was synthesised by Suzuki et al. [36]. The fractionation experiment was performed in a 1 m length and 8 mm inner diameter and 23.8 g dry high cross-linkage resin using lithium acetate and latter potassium acetate as eluent, while the isotope ratio of lithium in the eluted samples was analysed by Suzuki et al. [36]. Upon the experiment, a maximum value of isotope separation factor of 1.0066 was found, while the separation coefficient (ε = α − 1) was 0.0066 [36], values which show the potential of high cross-linkage resins in lithium isotope separation.

**Figure 3:**
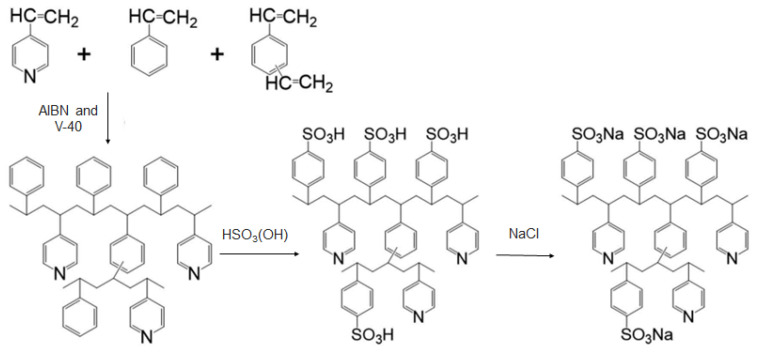
Synthesis of sulfonated pyridine-styrene-divinyl-benzene resin embedded into silica (Pyr-Styr-DVB/SiO_2_) for lithium isotope separation. Adapted from [38].

In another study, Putra et al. [37] investigated the lithium isotope separation in cation exchange resins characterized by 90% high cross-linkage degree obtained by adjusting the ratio between divinylbenzene and styrene to 9:1 during synthesis with suspension polymerization. The separation coefficient (ε) obtained in this study [37] was just 0.0069, slightly higher than the separation coefficient previously reported by Suzuki et al. [36] for a cross-linkage degree of 50%. Therefore, a correlation between the separation of lithium isotopes and cross-linkage degree was also investigated by Putra et al. [37] and the study concluded that saturation of the separation coefficient is attributable to the increase in hydrophobicity of the resin with increasing cross-linkage degree which may induce the so-called “dehydration effect”, the removal of lithium ions and hydration water molecules from the resin structure. More recently sulfonated pyridine-styrene-divinyl-benzene resin with a cross-linkage degree of 50% was first synthesized by Tachibana et al. by embedding the polymer into porous silica beads (thus abbreviated as sulfonated Pyr-Styr-DVB/SiO_2_), since the embedding can improve the stability (reduce the shrinking, as well the swelling, of the resin) and mechanical properties of the resin (see Figure 3) [38].

The values separation coefficient during lithium isotope fractionation of the above-mentioned resin compared with sulfonated styrene-divinyl-benzene (sulfonated Styr-DVB resin) had the same cross-linkage degree of 50% in batch experiments spanning a wide temperature range (278 to 333 K). The distribution coefficient values (K_d_) of Li ion in an aqueous solution were assessed and it was found that the K_d_ values of sulfonated Pyr-Styr-DVB/SiO_2_ resin were lower than those recorded for sulfonated Styr-DVB resin, and from this it can be concluded that pyridine moieties from the sulfonated Pyr-Styr-DVB/SiO_2_ have a small adsorption capacity for Li ions [38], possibly due to the lower aromatic character of pyridine which may decrease the π−π interactions/stackings [39,40]. The isotope separation coefficients (ε) per unit mass (ε/ΔMass) value of the sulfonated Pyr-Styr-DVB/SiO_2_ resin was calculated to 8.1 × 10^–4^ values, which is smaller than the ε/ΔMass values of many Styr-DVB resins (with cross-linkage degree of 50%) reported in the literature for lithium isotope separation studies [41]. Tachibana et al. conclude that the hydrophobicity of resins can improve the ε/ΔMass values, while those values are not proportional to the crosslinkage degree of the resins [38].

#### 2.3.3. Methods Involving Resin-Supported Complexing Agents

**Figure 4:**
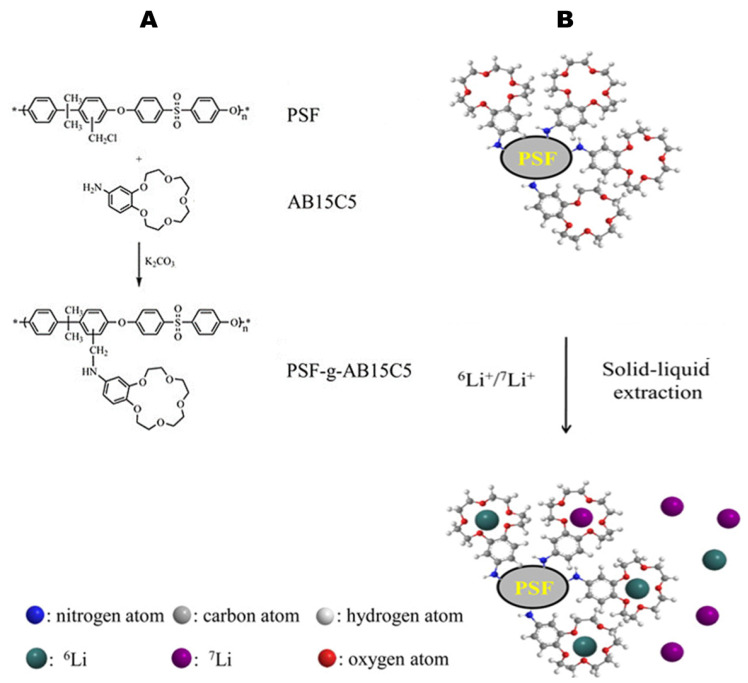
Synthesis route of crown ether-grafted polymer PSF-g-AB15C5 (**A**). Lithium isotope separation mechanism using PSF-g-AB15C5 (**B**). Adapted from [43].

The ion-exchange chromatography can also be performed with resin-supported complexing agents. In the past, [2_B_,1,1] cryptand resins have been packed in a column (9 mm ID and 220 mm long) to separate the Li isotopes. Separation factors α have been determined by Nishizawa et al. [42] for the partition of ^6^Li and ^7^Li between complexed ions with a cryptand [2_B_,2,1] resin, and lithium salts in methanol in a single-stage process. The study was conducted at different temperatures (0, 20 and 40 °C) and three Li halides (LiCl, LiBr, and LiI) were tested [42]. The separation factors α were calculated as the isotope ratio ^6^Li/^7^Li between the [2_B_,1,1] cryptand polymer and halides phase and decreased with temperature for all three Li halides, while the highest values were recorded at 0 °C: 1.045 ± 0.002 (LiI), 1.045 ± 0.003 (LiBr), and the highest, 1.047 ± 0.004 (LiCl) [42]. More recently, as an innovative method, a novel lithium isotope separation polymer polysulfone (PSF)-graft-4′-aminobenzo-15-crown-5-ether (PSF-g-AB15C5) was synthesised through a nucleophilic substitution mechanism by Yan et al. [43] from polysulfone (PSF) and 4′-aminobenzo-15-crown-5-ether (AB15C5) as starting materials for the synthesis (see Figure 4).

Then the partition of lithium isotope was investigated during batch experiments of solid–liquid extraction using the above-mentioned polymer [43] and it was found that ^6^Li, was enriched in the PSF-g-AB15C5 polymer. The effect of halide salts was also investigated, and in contrast with the findings of Nishizawa et al. [42], the order of the single stage separation factor obtained using different lithium salts in methanol decreased in the order LiCl < LiClO4 < LiBr < LiCI. Additionally, different solvents were tested and at the same concentration of crown ether AB15C5 of 0.51 mmol/g PSF-g-AB15C5 immobilized on polymers, the separation factors increased as 1.010 ± 0.002 (methanol) < 1.019 ± 0.002 (ethanol) < 1.021 ± 0.002 (propylene carbonate) < 1.027 ± 0.003 (acetonitrile), <1.031 ± 0.002 (nitromethane), according to the donicity of the solvents which represents a quantitative measure for the tendency of a solvent to donate electron pairs to acceptors [43]. With respect to crown ether concentration, the separation factor decreased from 1.015 ± 0.002 to 1.003 ± 0.001 with the decrease in AB15C5 concentration immobilized on polymer from 0.79 to 0.23 mmol/g in the CH_3_OH–LiCl/PSF-g-AB15C5 system [43].

#### 2.3.4. Methods Involving Ion-Exchange Membranes

Employing a lower working pressure, the membrane chromatography can provide a similar lithium isotope separation efficiency as column chromatography, but avoiding the intra-particle diffusions as a mass transfer-determining step. It is believed that the separation of lithium isotopes with membrane chromatography takes place by multilayer adsorption, surface diffusion, as well by ion–pore electrostatic interaction between Li ions, and the crown ether moieties on the membranes. In the case of membrane chromatography, the convective transport through membrane pores is the transfer-determining step. A PSF-g-AB15C5-type polymer porous membrane with porosity of 80.4% and average pore size of 62.7 nm was prepared by Pei et al. [44], while a concentration of 0.52 mmol immobilized AB15C5/g polymer was used. Discs with 24 mm-diameter were produced by cutting the PSF-g-AB15C5 membranes with an average thickness of 100 μm, while three layers of porous polyester (PET) filter were used as support between every two membranes (pore size of 300 μm and thickness of 200 μm), so a stationary phase packed in a chromatography column (Ø 25 × 100 mm) was built, resulting in a four-stage tandem membrane chromatography system [44]. In such a chromatographic system, the eluate depleted in ^6^Li obtained from the previous stage is used as the feed solution for the next stage [44]. The separation factor obtained from the single-stage membrane chromatography was up to 1.0232, while the relative abundances of ^6^Li in the four-stage tandem membrane chromatography increased by 0.2% (from 7.60 to 7.80%) [44].

### 2.4. Challenges in Development of Analysis Methods for Lithium Isotopes Using Spectrometric and Spectroscopic Methods

#### 2.4.1. Analysis Methods for Lithium Isotopes Using ICP-MS Methods

ICP-MS methods have been applied in various studies for Li isotope determination [45,46]. Many of these methods are using inductively coupled plasma-quadrupole mass spectrometry methods (ICP-Q-MS) [45].

The main challenges of the lithium isotope measurements with ICP-MS are changes of ionization equilibria in the plasma due to ionization of matrix interferences (like Na, Ca, and K). Thus, to reduce the matrix bias effects it is necessary to achieve quantitative separation of lithium from other interfering elements [32,47]. Nevertheless, because of the small separation factor of the Li^+^ and Na^+^ ions, the separation of lithium from sodium is difficult to achieve. As many clean-up methods are based on the ion exchange chromatography, small columns are the option of choice, due to the fraction of lithium isotopes on large columns [17]. Grégoire et al. used ICP-Q-MS to determine the lithium isotope ratios from minerals with a precision of ±0.8‰ (relative to reference material IRM-016 Li_2_CO_3_) [45]. Misra et al. determined lithium isotope ratios from seawater and naturally occurring carbonates, using chromatographic separation of lithium isotopes using a low volume-column filed with cation exchange resin (with 100–200 mesh size and total capacity of 3.4 meq) followed by ICP-Q-MS analysis [17]. The average lithium isotope composition of seawater was 30.75 ± 0.41‰ vs. L-SVEC (*n* = 10), while for the foraminifera carbonates the recorded isotope compositions were 30.72 ± 1.43 ‰ (*n* = 5) for coretop foraminifera from Caribbean Sea and 30.16 ± 1.37‰ (*n* = 4) for Gulf of Mexico foraminifera, respectively [17]. Therefore, it was concluded that a precision higher than ±1.5‰ vs. L-SVEC could be achieved for real samples [17]. Liu and Li optimized the determination of lithium isotope ratios from geological materials with ICP-Q-MS [48]. In this study, a higher precision of ±1.5‰ vs. L-SVEC was achieved in conditions of low whole-procedure blank (<0.004 ng) and high matrix tolerance (Na/Li ratio of 100), which allows for low mass consumption (2.5 ng of Li) [48].

Very recently, Juzer et al. developed an inductively coupled plasma-triple quadrupole mass method QQQ-ICP-MS [49]. Using a low volume column filed with cation exchange resin for chromatographic separation, Juzer et al. determined the lithium ratio of the seawater to 31.34 ± 0.56‰ (*n* = 49), which falls within the values determined previously by Misra et al. [17]. Comparing with ICP-Q-MS methods, this studied achieved a higher precision of ≤0.6‰ (2σ) at sub-ng levels of Li per sample [17]. Regarding the separation studies of lithium isotopes, ICP-Q-MS analysis has been used in few studies [36,38], although MC-ICP-MS methods were preferred.

#### 2.4.2. Analysis Methods for Lithium Isotopes Using MC-ICP-MS Methods

**Figure 5:**
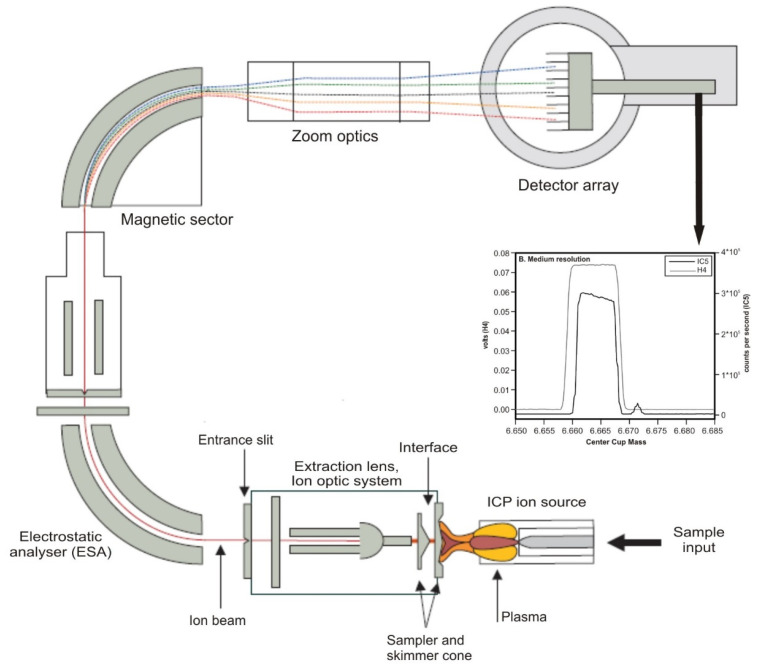
Schematic setup of an MC-ICP-MS with a magnetic sector analyser in Nier-Johnson geometry, used in lithium isotope analysis. Adapted from Ref. [54]. Adapted with permission from Ref. [55]. Copyright 2024 Elsevier.

Many studies are using inductively coupled plasma multicollector mass spectrometry (MC-ICP-MS) [50,51,52,53] to determine lithium isotope ratios from artificial and environmental samples (see Figure 5).

Comparing with ICP-Q-MS, the MC-ICP-MS methods appear to be more appropriate for low quantities of environmental samples. Nevertheless, historically, MC-ICP-MS methods had some drawbacks: a relatively high sensitivity to matrix interferences, mass bias due to matrix, the occurrence of lithium fractionation inside the instrument, and the requirement of a relatively high level of lithium into the sample (2 to 40 ng) [17,47].

Tomascak et al. were first to report reliable and precise measurements of Li isotopes with MC-ICP-MS [47]. This study determined the lithium isotope ratios from seawater and a basalt standard (JB-2) [47]. Upon digestion, as in the case of ICP-Q-MS, this study used cation exchange resin (AG50W-x8 type) to separate the lithium followed by MC-ICP-MS analysis, while the precision of the measurements was calculated to ±1.1‰ vs. L-SVEC [47]. Huang et al. performed an accurate determination in environmental materials with MC-ICP-MS but with a much higher precision of ±0.12‰ vs. L-SVEC (*n* = 46), while requiring just about 1.2 ng Li per sample [56]. Huang et al. used an optimised procedure with high chemical yields due to one-step column chromatographic separation involving lower procedure blanks (<5 pg) to record the lithium isotope ratios of two seawater references as ranged 30.88 ± 0.12‰ to 30.73 ± 0.15 vs. L-SVEC at a low mass resolution of 500 (valley definition of 5% peak height) [56]. Tian et al. [57] determined the isotope ratio of lithium in reference materials (andesite (AGV-2), basalt (BHVO-2) and IRMM-016 reference standard) using MC-ICP-MS. Prior to the MC-ICP-MS analysis, lithium was enriched in stages on three different columns (the first two eluted with HCl of different concentration and the 3rd with 30% ethanol) using synthetic cation-exchange resin [57]. The first stage removed the major cations from the matrix, the second stage removed the major elements except sodium, and the third stage removed the sodium ions from the matrices [57]. The precision of lithium isotope ratio determination with MC-ICP-MS was calculated ±0.72–1.04‰ (2σ), but overall, this study showed the complexity of sample preparation required for lithium isotope measurements in some materials [57]. In the last years, MC-ICP-MS, in combination with laser ablation (LA) (thus LA-MC-ICP-MS) became a popular technique for lithium isotope determination directly from solid phases, thus preserving spatial resolution, as this technique is capable of sampling a volume of >1 μm in depth and approximately 10–100 μm in diameter [58].

The main advantage of LA-MC-ICP-MS for lithium isotopes over classical MC-ICP-MS appears to be minimal sample preparation, but there are also other advantages such as good spatial resolution, low sample size, high sample throughput, and a low likelihood of contamination [58]. The main disadvantage of LA-MC-ICP-MS is the occurring isotopic fractionation during laser ablation [59,60,61], an effect that can be limited in the case of using femtosecond (fs) laser ablation (in contrast with nanosecond LA) by reducing the ablation crater in the sample to nm-sized diameter [62]. Some studies used fs-LA-MC-ICP-MS for *in situ* measurements of lithium isotope ratios. Steinmann et al. used a UV-femtosecond laser ablation coupled with MC-ICP-MS for *in situ* measurements of lithium isotope ratios from silicate reference glasses and olivines containing low levels of lithium (in μg/g range), and the precision has been calculated to about 2‰ (2σ) vs. L-SVEC [62]. In this study the isotope ratio values obtained with *in situ* measurements were also compared with those obtained with MC-ICP-MS performed in solutions (upon sample preparation) and they were in good agreement [62].

#### 2.4.3. Analysis Methods for Lithium Isotopes Using Thermal Ionisation Mass Spectrometry

Historically, the thermal ionisation mass spectrometry has been the most-used method for determination of lithium isotope ratios using various ionization methodologies [63,64,65,66,67].

Arienzo et al. [68] employed ion exchange chromatography based on cation exchange resin followed by thermal Ionization Mass Spectrometry (TIMS) to determine the ratio of Li isotopes for both natural and reference samples (the solid NIST L-SVEC and BHVO-2 standards and seawater) by using two different types of thermal ionization mass spectrometers configured in static (the magnetic field remains static) and dynamic (the magnetic field varies) modes. The determined isotope composition of BHVO-2 was 3.43‰ vs. L-SVEC, while for the seawater it ranged from 30.6 to 33.7‰ vs. L-SVEC [68], values which correspond to the reported literature values. More recently, Bhushan et al. [69] used a TIMS methodology called total evaporation and ion integration technique (TE and II). This technique involves complete evaporation of the lithium isotopes from the filament in the thermal ionisation source, and measuring of the ion beams of lithium isotopes until complete exhaustion of the sample [70]. Using this technique, the lithium isotope ratios were determined using the molecular ions *m*/*z* 71–73 (NaLiBO_2_^+^) and *m*/*z* 88–89 (Na_2_BO_2_^+^) emitted from the sodium lithium borate and a good precision of 0.05% was obtained [69]. Nevertheless, further studies are needed to elucidate the potential of total evaporation and ion integration techniques as alternative methodologies in lithium isotope determinations.

## 3. Conclusions

The most technically viable techniques presented in this review were two phase equilibrium system (using crown ethers), ion-exchange chromatography, and electromigration, with the latter being more technically challenging. Nevertheless, only the chemical equilibrium separation using crown ethers has given separation factors comparable to that of Li amalgam method. Electromigration and electrodialysis methodologies appear to be the most eco-friendly and promising alternatives for the separation of lithium isotopes, but the different experimental approaches must be optimised. Further experimental approaches using the electrochemical isotope effect are expected to appear in the next years. Displacement chromatography methods are some of the old, yet still used, methods for separation of lithium isotopes. Among them, the ion exchange method using the crown ethers grafted on polymers is one of the most innovative and studied methods. With respect to the lithium isotope analysis methods, the development of new ICP-Q-MS, MC-ICP-MS and TIMS methods first requires quantitative separation of lithium from other interfering elements. In this case, the ion exchange based on small columns is the main option, due to the fraction of lithium isotopes on large columns. The MC-ICP-MS methods gave a better precision in lithium isotope analysis than those of ICP-Q-MS. With respect to analyses of Li isotopes, further strategies based on inductively coupled plasma-high resolution mass spectrometry (ICP-HRMS) are expected to be developed.

## Figures and Tables

**Table 1:** The main advantages/disadvantages of the main groups of extraction methods.

Method Type	Main Advantages/Disadvantages			
	Separation Factor	Eco Friendly	Complexity of Methodology	Costs
Chemical exchange methods in two liquid phases	High	Very little	Low	Very high
Electrochemical exchange methods	Low	Moderate	High	Moderate
Displacement chromatography methods	Low	Moderate	Relatively low	Moderate

## Data Availability

Not applicable.
