# Correction: Badea et al. New Trends in Separation Techniques of Lithium Isotopes: A Review of Chemical Separation Methods. *Materials* 2023, *16*, 3817

**DOI:** 10.3390/ma18040807

**Published:** 2025-02-12

**Authors:** Silviu-Laurentiu Badea, Violeta-Carolina Niculescu, Andreea-Maria Iordache

**Affiliations:** National Research and Development Institute for Cryogenic and Isotopic Technologies, 4th Uzinei Street, 240050 Râmnicu Vâlcea, Romania

In the original publication [1], “Scott, S.R.; Shafer, M.M. A new method for measurement of lithium isotopes with nuclear applications. *Prog. Nucl. Energy* **2020**, *120*, 103234” was not cited in Figure 5. The citation has now been inserted in the caption of Figure 5. With this correction, the order of some references has been adjusted accordingly.

The authors state that the scientific conclusions are unaffected. This correction was approved by the Academic Editor. The original publication has also been updated.
